# RepeatCraft: a meta-pipeline for repetitive element de-fragmentation and annotation

**DOI:** 10.1093/bioinformatics/bty745

**Published:** 2018-08-25

**Authors:** Wai Yee Wong, Oleg Simakov

**Affiliations:** Department of Molecular Evolution and Development, Faculty of Life Science, University of Vienna, A-1090 Vienna, Austria

## Abstract

**Summary:**

Repetitive elements comprise large proportion of many genomes. They have impact on both genome evolution and regulation. Their classification and the study of evolutionary history is a major emerging field. Various software exist to-date to classify and map repeats across genomes. The major unresolved drawback, however, is the fragmented nature of many identified repeat loci. This ultimately makes the classification of novel repeats and their evolutionary analyses difficult. To improve on this, we developed a pipeline (RepeatCraft) that integrates results from several repeat element classification tools based on both sequence similarity and structural features. The pipeline de-fragments closely spaced repeat loci in the genomes, reconstructing longer copies, thus allowing for a better annotation and sequence comparisons. The pipeline also includes a user interface that can run in a web browser allowing for an easy access and exploration of the repeat data.

**Availability and implementation:**

RepeatCraft is implemented in Python and the web application is implemented in R. Download and documentation is freely available at https://github.com/niccw/repeatCraftp.

**Supplementary information:**

Supplementary data are available at *Bioinformatics* online.

## 1 Introduction

Repetitive elements comprise a large proportion of many metazoan genomes. Three major classes exist: DNA elements (cut and paste propagation), retroelement (copy paste propagation) and simple repeats (not autonomous). The length of individual repeat elements can vary from a few base pairs (simple repeats) to a dozen kilobases [e.g. helitrons (Kapitonov and Jurka, 2001)]. Active autonomous repeat elements contain enzymes that enable their propagation, e.g. transposase for DNA and reverse transcriptase for long interspersed nuclear elements (LINEs), respectively. Some elements harbour additional structural features such as the long terminal repeats (LTR) that facilitate their recognition and transcription as well as insertion into the genome.

Despite the prevalence in the genomes and many reports on their role in the evolution of gene regulation and genome stability (Feschotte and Pritham, 2007), detection and classification of repetitive elements remains problematic. Many unclassified repeat families are emerging in genomic studies (Chapman *et al.*, 2010; Chalopin *et al.*, 2014). One of the major problems in the classification is that the current tools of repeat annotation report fragmented pieces of transposon in the genomes that hamper both the classification by sequence similarity to known elements and through the lack of any detectable structural features. Very few tools exist to date that allow exploration and validation of raw RepeatMasker outputs [e.g. Bailly-Bechet *et al.* (2014)].

Here, we introduce a meta-approach, RepeatCraft, that combines several annotation pipelines and a merging algorithm to solve this problem by extending annotated pieces of transposons into larger repeat loci and reclassifying them based on those complete sequences. The method both reduces the complexity of repeat ‘fragments’ found in the genomes as well as facilitates detection and classification of novel repeat classes. Finally, we introduce a multi-functional web-based user interface that allows to explore repeat data with the help of sequence similarity and structural features.

## 2 Materials and methods

**Fig. 1.**
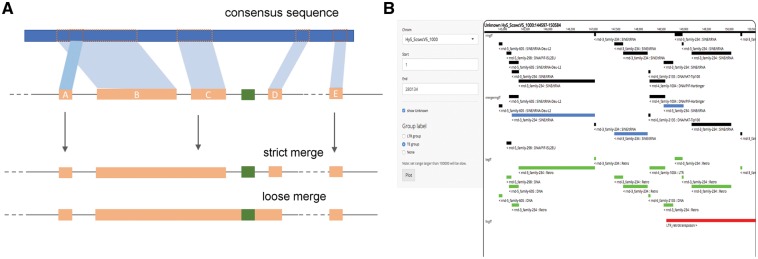
Schematic diagram (**A**) showing how RepeatCraft groups repeat ‘fragments’, based on their coverage in the consensus sequence (blue track) and the distance between consecutive repeats. By default, RepeatCraft only merges consecutive repeats (strict merging). The ‘loose’ merge considers non-consecutive closely spaced repeats and retains the annotation of other short repeats (i.e. simple repeats) in between the fragments. (**B**) The range plot panel of the web application provides a track-based visualization of the result of RepeatCraft, similar to a genome browser. The first track shows the annotation from RepeatMasker, the second track shows the improved annotation from RepeatCraft. The remaining tracks display the annotations from other tools (e.g. LTR_FINDER)

The pipeline is implemented in Python and builds off the coordinate file (in the genome feature format, GFF), as produced by any repeat masking tool, e.g. RepeatMasker (http://www.repeatmasker.org). The attribute field in the GFF file is reserved for the repeat family name and should also contain coordinates relative to the full-length or consensus sequence used in masking of the genome (as, e.g. in the default RepeatMasker output). In the first step, the pipeline updates the coordinate GFF file to populate and format the attribute column with annotations and coordinates on the reference sequence. Secondly, it identifies short (user-defined, by default 100 bp) repeats. Third step involves merging the output of other tools [currently, LTR_FINDER, (Xu and Wang, 2007)] labelling structural features (such as the LTRs, Fig. 1). In the final step, the sequence similarity and structural information is merged by the following rules. Neighbouring fragments of the same repeat element that are non-overlapping in the coverage of their corresponding consensus sequence are merged into and labelled as one locus. Combining structural evidence for each of those loci our pipeline updates their annotation. We implement two merging algorithms. Strict merging requires consecutive order of repeat fragments from the same family, without any intervening repeat of a different family [similar to Bailly-Bechet *et al.* (2014), yet also allowing for inversions]. ‘Loose’ merging additionally allows for such intervening families to be present provided that they fall within the maximum separation cutoff (default 150 bp). Many of such intervening repeat families comprise very short stretches of simple repeats and can be useful in the classification of the larger repeat loci they are embedded in.

## 3 Results

Running the pipeline with default parameters results in a reduction of some highly fragmented repeat loci. Based on a genome assembly of *Hydra magnipapillata* (https://research.nhgri.nih.gov/hydra) (Chapman *et al.*, 2010) we could reduce the total number of repeat loci as detected by RepeatMasker by around 10% over all repetitive elements (Supplementary Table S1), while not changing the overall repeat coverage. The highest level of repeat locus extension (fragment reduction) was observed for unknown repeats (around 60 000 merges). This further allowed us to assign structural features (e.g. ORFs ≥ of over 100 bp) to about 90% of previously unknown elements. Family-level annotation improvement statistics is provided in the Supplementary Tables S2–S4.

Finally, we designed a web application implemented in R as a graphical user interface of the RepeatCraft pipeline (Fig. 1), it also functions as an interactive tool for visualizing and browsing the repeat element annotation. It allows to study sequence similarity and structural annotation of the repeat families in a genome browser format, as well as on the individual family basis. Latter feature is of particular importance for the characterization of novel repeat elements whose longest loci can now be studied in terms of their structural features such as tandem repeats, LTRs and open reading frames.

## Funding

WYW and OS are supported by a grant from the Austrian Science Fund (FWF): P30686-B29.

*Conflict of Interest*: none declared.

## Supplementary Material

Supplementary Tables
