# Supplementary material for: RepeatCraft: a meta-pipeline for repetitive element de-fragmentation and annotation
Source: Bioinformatics. 2018 Aug 25;35(6):1051–2. doi: 10.1093/bioinformatics/bty745 (PMC6419915; doi:10.1093/bioinformatics/bty745)
Supplement: Supplementary Tables [file bty745_supplementary_tables.docx]

Table 1. Total number of repeat elements annotated by RepeatMasker v4.0.7, after strict merging and loose merging using RepeatCraft in *Hydra magnipapillata, Nematostella vectensis* (August 2014 JGI nemve1) and *Drosophila melanogaster* (March 2018 BDGP R6.22).

|  | Number of loci | | |
| --- | --- | --- | --- |
| Species | RepeatMasker | After Strict merging | After loose merging |
| *Hydra magnipapillata* | 1885452 | 1796244 | 1702943 |
| *Nematostella vectensis* | 507732 | 495441 | 472599 |
| *Drosophila melanogaster* | 144823 | 134650 | 131505 |

Table 2. Total number of repeat elements (by class) annotated by RepeatMasker v4.0.7, after strict after loose merge using RepeatCraft, percentage of merge of strict and loose merge and number of elements with TEgroup and/or LTRgroup label in strict and loose merging in *Hydra magnipapillata*.

|  | Number of loci | | | |  | Strict merge | | Loose merge | |
| --- | --- | --- | --- | --- | --- | --- | --- | --- | --- |
| Class | RepeatMasker | After Strict merging | % of merge | After  loose merging | % of merge | TEgroup | LTRgroup | TEgroup | LTRgroup |
| DNA | 12665 | 11973 | 5.46% | 11054 | 12.72% | 1328 |  | 3089 |  |
| DNA/Academ | 3215 | 2629 | 18.23% | 2403 | 25.26% | 1035 |  | 1415 |  |
| DNA/Academ-1 | 1337 | 1290 | 3.52% | 1235 | 7.63% | 92 |  | 197 |  |
| DNA/Academ-2 | 1925 | 1688 | 12.31% | 1588 | 17.51% | 441 |  | 612 |  |
| DNA/CMC-Chapaev | 34653 | 31898 | 7.95% | 30266 | 12.66% | 5063 |  | 8032 |  |
| DNA/CMC-Chapaev-3 | 9923 | 9241 | 6.87% | 8677 | 12.56% | 1318 |  | 2385 |  |
| DNA/CMC-EnSpm | 102642 | 98642 | 3.90% | 90408 | 11.92% | 7699 |  | 23562 |  |
| DNA/CMC-Transib | 38291 | 35927 | 6.17% | 33790 | 11.75% | 4398 |  | 8398 |  |
| DNA/Crypton | 10090 | 9950 | 1.39% | 9475 | 6.10% | 277 |  | 1213 |  |
| DNA/Crypton-H | 9360 | 9124 | 2.52% | 8722 | 6.82% | 469 |  | 1256 |  |
| DNA/Crypton-S | 369 | 315 | 14.63% | 292 | 20.87% | 101 |  | 143 |  |
| DNA/Crypton-V | 488 | 487 | 0.20% | 453 | 7.17% | 2 |  | 70 |  |
| DNA/Ginger | 10025 | 9500 | 5.24% | 8501 | 15.20% | 1007 |  | 2954 |  |
| DNA/hAT | 264 | 261 | 1.14% | 255 | 3.41% | 6 |  | 18 |  |
| DNA/hAT-Ac | 35667 | 32922 | 7.70% | 30566 | 14.30% | 5162 |  | 9593 |  |
| DNA/hAT-Blackjack | 3633 | 3094 | 14.84% | 2875 | 20.86% | 972 |  | 1345 |  |
| DNA/hAT-Charlie | 705 | 605 | 14.18% | 547 | 22.41% | 182 |  | 286 |  |
| DNA/hAT-hAT5 | 13142 | 12386 | 5.75% | 11792 | 10.27% | 1355 |  | 2449 |  |
| DNA/hAT-hATm | 59500 | 52320 | 12.07% | 49443 | 16.90% | 12975 |  | 17966 |  |
| DNA/hAT-hATw | 12530 | 11605 | 7.38% | 10996 | 12.24% | 1704 |  | 2838 |  |
| DNA/hAT-hATx | 13394 | 12067 | 9.91% | 11458 | 14.45% | 2487 |  | 3574 |  |
| DNA/hAT-hobo | 321 | 316 | 1.56% | 312 | 2.80% | 10 |  | 18 |  |
| DNA/hAT-Pegasus | 1290 | 909 | 29.53% | 839 | 34.96% | 665 |  | 775 |  |
| DNA/hAT-Tag1 | 5206 | 4474 | 14.06% | 4139 | 20.50% | 1318 |  | 1907 |  |
| DNA/hAT-Tip100 | 108486 | 100223 | 7.62% | 94068 | 13.29% | 15447 |  | 26783 |  |
| DNA/hAT-Tol2 | 484 | 404 | 16.53% | 379 | 21.69% | 144 |  | 185 |  |
| DNA/Kolobok | 14322 | 13578 | 5.19% | 12638 | 11.76% | 1443 |  | 3249 |  |
| DNA/Kolobok-Hydra | 10171 | 9390 | 7.68% | 8954 | 11.97% | 1479 |  | 2303 |  |
| DNA/Maverick | 12711 | 11593 | 8.80% | 10700 | 15.82% | 2052 |  | 3675 |  |
| DNA/Merlin | 3813 | 3566 | 6.48% | 3286 | 13.82% | 459 |  | 983 |  |
| DNA/MuLE-MuDR | 1366 | 1150 | 15.81% | 1107 | 18.96% | 397 |  | 468 |  |
| DNA/MULE-MuDR | 1161 | 1097 | 5.51% | 1025 | 11.71% | 397 |  | 468 |  |
| DNA/MuLE-NOF | 207 | 200 | 3.38% | 197 | 4.83% | 13 |  | 19 |  |
| DNA/P | 40201 | 37600 | 6.47% | 35491 | 11.72% | 4859 |  | 8772 |  |
| DNA/PIF-Harbinger | 2065 | 1762 | 14.67% | 1647 | 20.24% | 557 |  | 756 |  |
| DNA/PIF-ISL2EU | 11041 | 9730 | 11.87% | 9141 | 17.21% | 2337 |  | 3355 |  |
| DNA/PiggyBac | 1228 | 973 | 20.77% | 939 | 23.53% | 460 |  | 516 |  |
| DNA/Sola | 14511 | 12655 | 12.79% | 12053 | 16.94% | 3391 |  | 4435 |  |
| DNA/Sola-1 | 4504 | 4127 | 8.37% | 3842 | 14.70% | 687 |  | 1216 |  |
| DNA/Sola-2 | 22638 | 21264 | 6.07% | 20234 | 10.62% | 2642 |  | 4578 |  |
| DNA/Sola-3 | 2838 | 2644 | 6.84% | 2469 | 13.00% | 376 |  | 712 |  |
| DNA/TcMar-Fot1 | 42096 | 38303 | 9.01% | 36323 | 13.71% | 7076 |  | 10564 |  |
| DNA/TcMar-ISRm11 | 10201 | 9490 | 6.97% | 8425 | 17.41% | 1339 |  | 3423 |  |
| DNA/TcMar-Mariner | 3339 | 2983 | 10.66% | 2896 | 13.27% | 687 |  | 848 |  |
| DNA/TcMar-Pogo | 3303 | 2946 | 10.81% | 2785 | 15.68% | 670 |  | 958 |  |
| DNA/TcMar-Tc1 | 35013 | 31757 | 9.30% | 29664 | 15.28% | 6202 |  | 10038 |  |
| DNA/TcMar-Tc2 | 6401 | 6064 | 5.26% | 5960 | 6.89% | 637 |  | 832 |  |
| DNA/TcMar-Tigger | 314 | 282 | 10.19% | 271 | 13.69% | 63 |  | 84 |  |
| DNA/Zator | 15862 | 15207 | 4.13% | 13774 | 13.16% | 1210 |  | 4014 |  |
| DNA/Zisupton | 787 | 764 | 2.92% | 646 | 17.92% | 46 |  | 276 |  |
| LINE | 1873 | 1701 | 9.18% | 1631 | 12.92% | 328 |  | 454 |  |
| LINE/CR1 | 38422 | 36683 | 4.53% | 34903 | 9.16% | 3343 |  | 6779 |  |
| LINE/CR1-Zenon | 82749 | 75949 | 8.22% | 72367 | 12.55% | 12865 |  | 19372 |  |
| LINE/CRE | 369 | 369 | 0.00% | 347 | 5.96% | 0 |  | 44 |  |
| LINE/CRE-II | 538 | 458 | 14.87% | 422 | 21.56% | 149 |  | 213 |  |
| LINE/L1-Tx1 | 3158 | 2957 | 6.36% | 2795 | 11.49% | 380 |  | 685 |  |
| LINE/L2 | 112832 | 109659 | 2.81% | 103472 | 8.30% | 6196 |  | 18243 |  |
| LINE/L2-Hydra | 4622 | 4477 | 3.14% | 4287 | 7.25% | 281 |  | 655 |  |
| LINE/Penelope | 40113 | 38383 | 4.31% | 35164 | 12.34% | 3387 |  | 9646 |  |
| LINE/Proto2 | 2124 | 1928 | 9.23% | 1849 | 12.95% | 373 |  | 516 |  |
| LINE/Rex-Babar | 113 | 113 | 0.00% | 113 | 0.00% | 0 |  | 0 |  |
| LINE/RTE-BovB | 4087 | 3943 | 3.52% | 3799 | 7.05% | 279 |  | 551 |  |
| Low_complexity | 61341 | 61339 | 0.00% | 59521 | 2.97% | 4 |  | 3636 |  |
| LTR | 648 | 577 | 10.96% | 494 | 23.77% | 135 | 29 | 295 | 29 |
| LTR/Copia | 443 | 347 | 21.67% | 321 | 27.54% | 167 | 32 | 209 | 32 |
| LTR/DIRS | 441 | 396 | 10.20% | 340 | 22.90% | 78 | 12 | 188 | 12 |
| LTR/Gypsy | 16798 | 14891 | 11.35% | 13970 | 16.84% | 2634 | 1094 | 4525 | 1094 |
| LTR/Pao | 2965 | 2388 | 19.46% | 2281 | 23.07% | 815 | 278 | 1026 | 278 |
| Other/DNA_virus | 291 | 220 | 24.40% | 206 | 29.21% | 125 |  | 146 |  |
| RC/Helitron | 62213 | 60213 | 3.21% | 56329 | 9.46% | 3909 |  | 11345 |  |
| Satellite | 998 | 990 | 0.80% | 952 | 4.61% | 16 |  | 92 |  |
| Simple_repeat | 373703 | 373636 | 0.02% | 366342 | 1.97% | 133 |  | 14705 |  |
| SINE/tRNA | 41143 | 41022 | 0.29% | 40216 | 2.25% | 242 |  | 1850 |  |
| Unknown | 283768 | 270229 | 4.77% | 251822 | 11.26% | 26077 |  | 60857 |  |

Table 3. Total number of repeat elements (by class) annotated by RepeatMasker v4.0.7, after strict after loose merge using RepeatCraft, percentage of merge of strict and loose merge and number of elements with TEgroup and/or LTRgroup label in strict and loose merging in *Nematostella vectensis*.

|  | Number of loci | | | |  | | Strict merge | | | Loose merge | | |
| --- | --- | --- | --- | --- | --- | --- | --- | --- | --- | --- | --- | --- |
| Class | RepeatMasker | After Strict merging | % of merge | After  loose merging | | % of merge | | TEgroup | LTRgroup | | TEgroup | LTRgroup |
| DNA | 113353 | 110627 | (2.40%) | 105543 | | (6.89%) | | 5330 |  | | 15376 |  |
| DNA/Academ | 553 | 503 | (9.04%) | 472 | | (14.65%) | | 93 |  | | 147 |  |
| DNA/Academ-1 | 4211 | 4071 | (3.32%) | 3774 | | (10.38%) | | 277 |  | | 856 |  |
| DNA/Academ-2 | 4992 | 4862 | (2.60%) | 4111 | | (17.65%) | | 252 |  | | 1743 |  |
| DNA/CMC-Chapaev | 217 | 206 | (5.07%) | 198 | | (8.76%) | | 22 |  | | 36 |  |
| DNA/CMC-EnSpm | 4679 | 4601 | (1.67%) | 4321 | | (7.65%) | | 153 |  | | 703 |  |
| DNA/Crypton | 260 | 226 | (13.08%) | 208 | | (20.00%) | | 59 |  | | 92 |  |
| DNA/Crypton-A | 581 | 576 | (0.86%) | 558 | | (3.96%) | | 10 |  | | 46 |  |
| DNA/Crypton-H | 3009 | 3005 | (0.13%) | 2748 | | (8.67%) | | 8 |  | | 518 |  |
| DNA/Ginger | 422 | 358 | (15.17%) | 365 | | (13.51%) | | 128 |  | | 113 |  |
| DNA/hAT | 571 | 549 | (3.85%) | 497 | | (12.96%) | | 44 |  | | 148 |  |
| DNA/hAT-Ac | 1277 | 1231 | (3.60%) | 1123 | | (12.06%) | | 89 |  | | 301 |  |
| DNA/hAT-Blackjack | 3099 | 2915 | (5.94%) | 2827 | | (8.78%) | | 352 |  | | 504 |  |
| DNA/hAT-Charlie | 41 | 41 | (0.00%) | 40 | | (2.44%) | | 0 |  | | 2 |  |
| DNA/hAT-hAT1 | 341 | 318 | (6.74%) | 316 | | (7.33%) | | 45 |  | | 49 |  |
| DNA/hAT-hAT5 | 7193 | 7031 | (2.25%) | 6863 | | (4.59%) | | 285 |  | | 618 |  |
| DNA/hAT-hAT6 | 908 | 872 | (3.96%) | 843 | | (7.16%) | | 71 |  | | 129 |  |
| DNA/hAT-Tip100 | 15511 | 15359 | (0.98%) | 15089 | | (2.72%) | | 303 |  | | 842 |  |
| DNA/Kolobok | 1814 | 1730 | (4.63%) | 1654 | | (8.82%) | | 165 |  | | 317 |  |
| DNA/Kolobok-Hydra | 195 | 174 | (10.77%) | 169 | | (13.33%) | | 42 |  | | 51 |  |
| DNA/Kolobok-T2 | 30627 | 30035 | (1.93%) | 29262 | | (4.46%) | | 1160 |  | | 2683 |  |
| DNA/Maverick | 9437 | 8864 | (6.07%) | 8275 | | (12.31%) | | 1046 |  | | 2161 |  |
| DNA/MULE-F | 348 | 322 | (7.47%) | 289 | | (16.95%) | | 52 |  | | 118 |  |
| DNA/MULE-MuDR | 3105 | 3036 | (2.22%) | 2896 | | (6.73%) | | 137 |  | | 413 |  |
| DNA/MULE-NOF | 748 | 710 | (5.08%) | 678 | | (9.36%) | | 76 |  | | 140 |  |
| DNA/PIF-Harbinger | 27845 | 27128 | (2.57%) | 25617 | | (8.00%) | | 1407 |  | | 4387 |  |
| DNA/PIF-ISL2EU | 97 | 86 | (11.34%) | 84 | | (13.40%) | | 21 |  | | 25 |  |
| DNA/PiggyBac | 7262 | 6993 | (3.70%) | 6848 | | (5.70%) | | 533 |  | | 817 |  |
| DNA/Sola | 1240 | 1231 | (0.73%) | 1182 | | (4.68%) | | 18 |  | | 112 |  |
| DNA/Sola-3 | 1787 | 1767 | (1.12%) | 1726 | | (3.41%) | | 39 |  | | 121 |  |
| DNA/TcMar-ISRm11 | 1062 | 1052 | (0.94%) | 1025 | | (3.48%) | | 20 |  | | 74 |  |
| DNA/TcMar-Pogo | 3433 | 3400 | (0.96%) | 3233 | | (5.83%) | | 64 |  | | 397 |  |
| DNA/TcMar-Tc1 | 19 | 18 | (5.26%) | 16 | | (15.79%) | | 2 |  | | 6 |  |
| DNA/TcMar-Tc2 | 10540 | 10378 | (1.54%) | 10101 | | (4.17%) | | 323 |  | | 872 |  |
| DNA/TcMar-Tigger | 1396 | 1334 | (4.44%) | 1276 | | (8.60%) | | 123 |  | | 237 |  |
| DNA/Zator | 462 | 435 | (5.84%) | 418 | | (9.52%) | | 54 |  | | 87 |  |
| LINE | 99 | 99 | (0.00%) | 98 | | (1.01%) | | 0 |  | | 2 |  |
| LINE/CR1 | 2763 | 2683 | (2.90%) | 2617 | | (5.28%) | | 156 |  | | 280 |  |
| LINE/CRE | 59 | 58 | (1.69%) | 56 | | (5.08%) | | 2 |  | | 6 |  |
| LINE/CRE-II | 116 | 100 | (13.79%) | 96 | | (17.24%) | | 31 |  | | 39 |  |
| LINE/I-Jockey | 369 | 369 | (0.00%) | 352 | | (4.61%) | | 0 |  | | 34 |  |
| LINE/L1-Tx1 | 295 | 293 | (0.68%) | 289 | | (2.03%) | | 4 |  | | 12 |  |
| LINE/L2 | 4327 | 4234 | (2.15%) | 4067 | | (6.01%) | | 183 |  | | 511 |  |
| LINE/Penelope | 13618 | 13296 | (2.36%) | 12111 | | (11.07%) | | 636 |  | | 2962 |  |
| LINE/Rex-Babar | 2115 | 2057 | (2.74%) | 2002 | | (5.34%) | | 107 |  | | 217 |  |
| LINE/RTE-BovB | 1067 | 1026 | (3.84%) | 1012 | | (5.15%) | | 79 |  | | 106 |  |
| LINE/RTE-X | 1656 | 1607 | (2.96%) | 1578 | | (4.71%) | | 98 |  | | 153 |  |
| Low_complexity | 6609 | 6609 | (0.00%) | 6509 | | (1.51%) | | 0 |  | | 200 |  |
| LTR | 2421 | 2239 | (7.52%) | 2145 | | (11.40%) | | 235 | 216 | | 428 | 216 |
| LTR/Copia | 1133 | 1064 | (6.09%) | 1040 | | (8.21%) | | 52 | 90 | | 100 | 90 |
| LTR/DIRS | 701 | 611 | (12.84%) | 570 | | (18.69%) | | 140 | 54 | | 216 | 54 |
| LTR/Gypsy | 9627 | 8642 | (10.23%) | 8176 | | (15.07%) | | 545 | 1637 | | 1548 | 1637 |
| LTR/Gypsy-Troyka | 292 | 224 | (23.29%) | 219 | | (25.00%) | | 91 | 50 | | 99 | 50 |
| LTR/Ngaro | 1408 | 1224 | (13.07%) | 1198 | | (14.91%) | | 179 | 246 | | 248 | 246 |
| LTR/Pao | 819 | 685 | (16.36%) | 629 | | (23.20%) | | 120 | 173 | | 234 | 173 |
| RC/Helitron | 22901 | 22477 | (1.85%) | 21616 | | (5.61%) | | 812 |  | | 2478 |  |
| rRNA | 2146 | 2139 | (0.33%) | 1949 | | (9.18%) | | 14 |  | | 394 |  |
| Satellite | 13144 | 13008 | (1.03%) | 11986 | | (8.81%) | | 268 |  | | 2264 |  |
| Simple_repeat | 52045 | 52036 | (0.02%) | 51279 | | (1.47%) | | 18 |  | | 1530 |  |
| SINE? | 4382 | 4122 | (5.93%) | 3811 | | (13.03%) | | 487 |  | | 1052 |  |
| SINE/tRNA-Deu | 2695 | 2685 | (0.37%) | 2594 | | (3.75%) | | 20 |  | | 202 |  |
| SINE/tRNA-Deu-L2 | 2496 | 2467 | (1.16%) | 2368 | | (5.13%) | | 56 |  | | 247 |  |
| SINE/tRNA-L2 | 2036 | 2031 | (0.25%) | 1904 | | (6.48%) | | 10 |  | | 264 |  |
| SINE/tRNA-Rex | 863 | 863 | (0.00%) | 770 | | (10.78%) | | 0 |  | | 186 |  |
| SINE/tRNA-V | 1644 | 1612 | (1.95%) | 1529 | | (7.00%) | | 64 |  | | 230 |  |
| snRNA | 97 | 95 | (2.06%) | 92 | | (5.15%) | | 4 |  | | 10 |  |
| Unknown | 91150 | 88740 | (2.64%) | 83290 | | (8.62%) | | 4677 |  | | 15410 |  |

Table 4. Total number of repeat elements (by class) annotated by RepeatMasker v4.0.7, after strict after loose merge using RepeatCraft, percentage of merge of strict and loose merge and number of elements with TEgroup and/or LTRgroup label in strict and loose merging in *Drosophila melanogaster*.

|  | Number of loci | | | |  | Strict merge | | Loose merge | |
| --- | --- | --- | --- | --- | --- | --- | --- | --- | --- |
| Class | RepeatMasker | After Strict merging | % of merge | After  loose merging | % of merge | TEgroup | LTRgroup | TEgroup | LTRgroup |
| ARTEFACT | 1 | 1 | (0.00%) | 1 | (0.00%) | 0 |  | 0 |  |
| DNA | 103 | 86 | (16.50%) | 85 | (17.48%) | 34 |  | 36 |  |
| DNA?/hAT? | 12 | 12 | (0.00%) | 12 | (0.00%) | 0 |  | 0 |  |
| DNA/CMC-Chapaev-3 | 1 | 1 | (0.00%) | 1 | (0.00%) | 0 |  | 0 |  |
| DNA/CMC-Transib | 1022 | 806 | (21.14%) | 781 | (23.58%) | 350 |  | 390 |  |
| DNA/hAT | 6 | 6 | (0.00%) | 5 | (16.67%) | 0 |  | 2 |  |
| DNA/hAT-Ac | 627 | 523 | (16.59%) | 517 | (17.54%) | 162 |  | 170 |  |
| DNA/hAT-Charlie | 3 | 3 | (0.00%) | 2 | (33.33%) | 0 |  | 2 |  |
| DNA/hAT-hobo | 319 | 233 | (26.96%) | 213 | (33.23%) | 152 |  | 188 |  |
| DNA/hAT-Pegasus | 134 | 132 | (1.49%) | 129 | (3.73%) | 4 |  | 10 |  |
| DNA/hAT-Tip100 | 3 | 3 | (0.00%) | 3 | (0.00%) | 0 |  | 0 |  |
| DNA/hAT? | 88 | 88 | (0.00%) | 88 | (0.00%) | 0 |  | 0 |  |
| DNA/Maverick | 116 | 83 | (28.45%) | 78 | (32.76%) | 53 |  | 59 |  |
| DNA/MULE-NOF | 61 | 55 | (9.84%) | 52 | (14.75%) | 10 |  | 16 |  |
| DNA/P | 2442 | 2071 | (15.19%) | 1993 | (18.39%) | 666 |  | 809 |  |
| DNA/PIF-Harbinger | 72 | 50 | (30.56%) | 46 | (36.11%) | 42 |  | 50 |  |
| DNA/PiggyBac | 25 | 18 | (28.00%) | 18 | (28.00%) | 11 |  | 11 |  |
| DNA/TcMar-Mariner | 25 | 22 | (12.00%) | 21 | (16.00%) | 6 |  | 8 |  |
| DNA/TcMar-Pogo | 74 | 56 | (24.32%) | 56 | (24.32%) | 34 |  | 34 |  |
| DNA/TcMar-Tc1 | 1280 | 996 | (22.19%) | 943 | (26.33%) | 514 |  | 611 |  |
| DNA/Zator | 1 | 1 | (0.00%) | 1 | (0.00%) | 0 |  | 0 |  |
| LINE/CR1 | 1605 | 1042 | (35.08%) | 1006 | (37.32%) | 925 |  | 978 |  |
| LINE/I | 452 | 342 | (24.34%) | 329 | (27.21%) | 184 |  | 204 |  |
| LINE/I-Jockey | 7618 | 6352 | (16.62%) | 6001 | (21.23%) | 2151 |  | 2766 |  |
| LINE/L2 | 4 | 4 | (0.00%) | 4 | (0.00%) | 0 |  | 0 |  |
| LINE/Penelope | 1 | 1 | (0.00%) | 1 | (0.00%) | 0 |  | 0 |  |
| LINE/R1 | 1607 | 1044 | (35.03%) | 1018 | (36.65%) | 887 |  | 929 |  |
| LINE/R1-LOA | 414 | 251 | (39.37%) | 242 | (41.55%) | 274 |  | 284 |  |
| LINE/R2 | 55 | 51 | (7.27%) | 51 | (7.27%) | 8 |  | 8 |  |
| LINE/RTE-BovB | 3 | 3 | (0.00%) | 3 | (0.00%) | 0 |  | 0 |  |
| LINE/RTE-X | 2 | 2 | (0.00%) | 2 | (0.00%) | 0 |  | 0 |  |
| Low_complexity | 10329 | 10328 | (0.01%) | 10137 | (1.86%) | 2 |  | 384 |  |
| LTR/Copia | 1275 | 952 | (25.33%) | 927 | (27.29%) | 395 | 172 | 440 | 172 |
| LTR/Gypsy | 18589 | 14114 | (24.07%) | 13374 | (28.05%) | 6562 | 1378 | 7853 | 1378 |
| LTR/Pao | 4134 | 2973 | (28.08%) | 2724 | (34.11%) | 1645 | 541 | 2046 | 541 |
| Other | 359 | 338 | (5.85%) | 318 | (11.42%) | 42 |  | 82 |  |
| RC/Helitron | 6648 | 6348 | (4.51%) | 6107 | (8.14%) | 594 |  | 1061 |  |
| RNA | 100 | 100 | (0.00%) | 100 | (0.00%) | 0 |  | 0 |  |
| rRNA | 156 | 145 | (7.05%) | 122 | (21.79%) | 20 |  | 63 |  |
| Satellite | 1809 | 1798 | (0.61%) | 1654 | (8.57%) | 22 |  | 307 |  |
| Simple_repeat | 82280 | 82279 | (0.00%) | 81442 | (1.02%) | 2 |  | 1675 |  |
| tRNA | 57 | 57 | (0.00%) | 57 | (0.00%) | 0 |  | 0 |  |
| Unknown | 910 | 881 | (3.19%) | 841 | (7.58%) | 57 |  | 137 |  |
